# Personalised care and support planning in Singapore: qualitative interviews with people living with diabetes

**DOI:** 10.3399/BJGPO.2023.0055

**Published:** 2024-03-06

**Authors:** Monica Lazarus, Tong Wei Yew, Wee Hian Tan, Kavita Venkataraman, Jose Maria Valderas, Doris Yee Ling Young, E Shyong Tai, Victor Weng Keong Loh

**Affiliations:** 1 Department of Medicine, Yong Loo Lin School of Medicine, National University of Singapore (NUS), Singapore, Singapore; 2 Department of Medicine, National University Hospital (NUH), Singapore, Singapore; 3 National University Polyclinics (NUP), Singapore, Singapore; 4 Saw Swee Hock School of Public Health, National University of Singapore (NUS), Singapore, Singapore; 5 Department of Family Medicine, National University Health System (NUHS), Singapore, Singapore

**Keywords:** diabetes mellitus, personalised care and support planning, primary health care, qualitative research

## Abstract

**Background:**

Personalised care and support planning (CSP) is a person-centred approach for the care of people living with long-term conditions. Patient Activation through Community Empowerment/Engagement for Diabetes Management (PACE-D) adapts the Year of Care Partnerships (YOCP) approach to CSP in the UK for people living with diabetes at Singapore polyclinics. Polyclinics are multi-storey primary care hubs that provide affordable, multidisciplinary, comprehensive, and high-throughput public health care for the multi-ethnic, multilingual Singapore population.

**Aim:**

To explore the experience of PACE-D-enrolled people living with diabetes with personalised CSP at Singapore polyclinics.

**Design & setting:**

Qualitative interviews of people living with diabetes who experienced personalised CSP at National University Polyclinics (NUP) in Singapore between July 2020 and November 2021.

**Method:**

PACE-D-enrolled people living with diabetes who experienced personalised CSP were purposively sampled. In-depth semi-structured interviews were recorded, transcribed, and analysed using Braun and Clarke’s reflexive thematic analysis.

**Results:**

Fifty-two patients participated in the study. Four main themes were identified. Theme 1 was the importance of the care-planning letter. Patients reported that the CPL prompted reflection and patient preparation for CSP conversations. Theme 2 was the role of the programme coordinator. PACE-D programme coordinators amplified self-management by playing advocate and confidant beyond administrative duties. Theme 3 was the value of the personalised CSP conversation. CSP providers were perceived as partners in care, with more time to listen compared with usual consultations. Patient engagement was affected by language confidence. Theme 4 was agency in self-management. With adequate time and support, patients increased in confidence and agency both in CSP engagement and diabetes self-management.

**Conclusion:**

While language confidence may affect patient engagement, personalised CSP shows promise for strengthening patient engagement and self-management among people living with diabetes at Singapore polyclinics.

## How this fits in

Active engagement and personal self-management are essential for patients to live well with diabetes and other long-term conditions. The Year of Care Partnerships (YOCP) person-centred approach to personalised care and support planning (CSP) reframes the purpose of care from *‘helping the person to manage their condition’* to *‘helping the person to live well with their condition’*.^1^ This is one of the first few studies to investigate the patient experience of CSP beyond the UK. Conducted at the Singapore polyclinics, this research has shown that personalised CSP is generally well received and promises to be an effective means to strengthen patient engagement and self-management by people learning to live well with their diabetes in a multicultural Asian primary care context.

## Introduction

Primary health care is globally challenged to provide efficient, effective, personalised care for the burgeoning demographic of people living with long-term conditions (LTCs).^2–4^ Personalised care and support planning (CSP) or the *'anticipatory negotiated discussion or series of discussions between a patient and a health professional to clarify goals, options, and preferences and develop an agreed plan of action based on this mutual understanding'* ^5^ is a theory-informed, person-centred approach that engages patients to live well with their diabetes and other LTCs. With decades of iteration in the UK, the Year of Care Partnerships (YOCP) approach to personalised CSP reframes the purpose of LTC care from *‘helping the person to manage their condition’* to *‘helping the person to live well with their condition’*.^1^

With a geographical location in the World Health Organization (WHO) Western Pacific that is projected to be the global epicentre of diabetes for the next century,^6^ Singapore’s multi-ethnic residents are a sociocultural *albeit* English-speaking microcosm of the region. With alarming rates of diabetes (Singapore prevalence 14.9%, UK prevalence 8.2%, global prevalence 10.9%),^6,7^ and a national 'War on Diabetes'(2016),^8^ Patient Activation through Community Empowerment/Engagement for Diabetes Management (PACE-D) adapts the person-centred YOCP approach to personalised CSP beyond the UK to the multicultural, multilingual public primary care setting at Singapore polyclinics.^9,10^ Designed for accessible, comprehensive, and efficient primary care, Singapore polyclinics are high-throughput, multidisciplinary primary care hubs with in-house laboratory and radiological services. Healthcare providers are organised into teamlets comprising two family physicians, one nursing care manager, one care coordinator, and an occasional clinical pharmacist,^11^ with each teamlet providing care for approximately 5000 empanelled patients.^11–14^ Time and resource constraints^15^ hinder providers' explorations of patients' intrinsic goals and concerns,^16^ often leaving providers little opportunity to actively support patients in what matters most in living well with their diabetes, based on their authentic goals and values.

**Figure 1.**
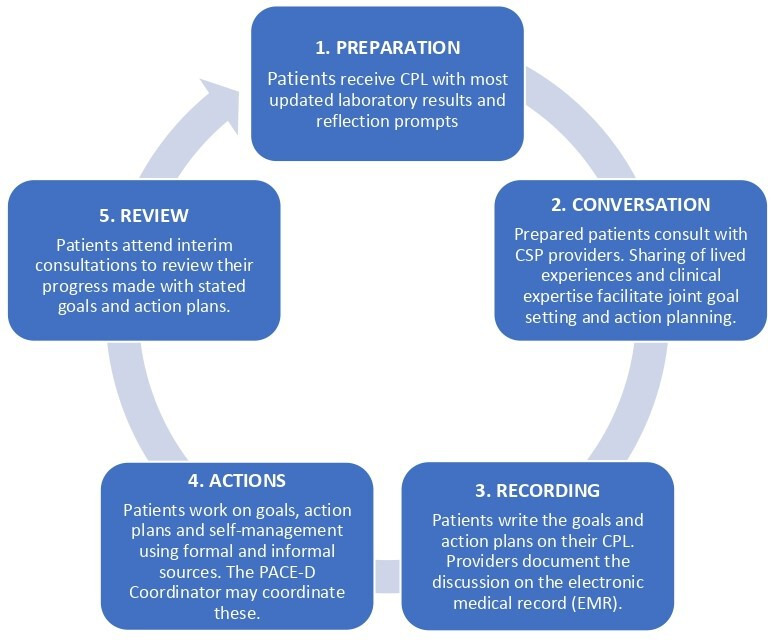
The process of personalised care and support planning (CSP) in Patient Activation through Community Empowerment/Engagement for Diabetes Management (PACE-D).^6^ CPL = care-planning letter.

The elements of PACE-D have been described elsewhere (Figure 1).^10^ Preparation of patients starts with the early receipt of the care-planning letter (CPL). As advised by patients in focus group discussions convened for the operation's pilot, the CPL takes the form of a handy A5-sized booklet that charts patients’ latest investigation results, including HbA1C as colourful infographics, with spaces intentionally left blank for enrolled patients to pen their thoughts (Supplementary Box S1). An extended 20–30-minute timeslot for each personalised CSP conversation instead of a 'usual' 10–12-minute consultation is embedded within the healthcare system delivery process. Consistent with evidence on the positive effects of person-centredness on self-management and health outcomes,^1,17^ trained providers intentionally explore patient goals so that jointly made decisions respectful of patients’ values may be made. Actionable goals are recorded on the CPL as reminders for patients and documented in the medical records for subsequent review by providers. Actions on decisions may range from mutually agreed-on weight-loss goals to attending socially prescribed activities linked by the PACE-D programme coordinator. Patients are given appointments for interim consultations to review their progress until their next annual personalised CSP.

While evidence of improved disease outcomes and better patient^9^ experience have been garnered for more than a decade in the UK, the novelty of this article is that we study the experience of the linguistically and culturally diverse Singapore primary care patient population with regards personalised CSP. Nested in the PACE-D main evaluation, this study will inform the main evaluation, and lend insight into how personalised CSP may be optimised for further implementation in Singapore primary care^10^ and possibly beyond Singapore.

## Method

Semi-structured interviews were conducted between July 2020 and November 2021 by two research trained non-clinical interviewers (ML and ASL). We recruited people living with diabetes who underwent personalised CSP with PACE-D teamlets at Pioneer and Jurong Polyclinics of the National University Polyclinics (NUP) cluster. Participants were purposively sampled for diversity in terms of age, sex, and ethnic group.^18^ A preliminary topic guide was developed from our literature review^19^ with input from key opinion leaders and the study team, and then piloted to assess suitability of questions (Supplementary Table S1).

Study participants provided written informed consent. There were 55 initial participants; three interviews were removed owing to poor quality data (for example, difficulty in understanding participant and/or background noise or conversation not recorded), resulting in a final sample of 52. Of these, 46 were interviewed in English, three in Malay, and three in Mandarin. Fifteen interviews were conducted in-person at the study sites, 34 via phone, and three via Zoom amid COVID-19 restrictions. Study participants received SGD50 (approximately GBP30) for participation. Interviews were conducted iteratively, with field notes taken after each interview. Interviews lasted 26–85 minutes, were audio-recorded, transcribed verbatim, and de-identified before analysis. Non-English interviews were translated by native speakers before analysis.

Data were analysed concurrently with collection. Analysis was guided by Braun and Clarke’s reflexive thematic analysis framework: familiarisation, generation of codes, searching for themes, reviewing of themes, and defining themes.^20^ Interviews were transcribed on Microsoft Word and imported onto Microsoft Excel, and organised for coding.^21^ Analysis was initially conducted inductively to have a comprehensive understanding of the collected data, before moving to a more deductive nature in order to map the themes and codes to the overall process of personalised CSP as used in the Singapore polyclinics (Figure 1).

Three researchers (ML, research associate; VL, CSP practitioner and family physician; and KV, primary care physician and researcher) developed the codebook iteratively to guide analysis. ML read and coded all interview transcripts. VL and KV read several transcripts independently and reviewed the codes. To mitigate any risk of bias from having two interviewers (ML and ASL), we checked and found that codes and themes identified were not disproportionately attributed to the transcripts of any particular interviewer.

To ensure reflexivity, interviewers explained their backgrounds to participants before interviews and kept a reflexivity journal. The multidisciplinary research team (primary care, public health, and endocrinology) regularly met to critically debrief, reflect on underlying assumptions, and discuss the research data to attain consensus in the codes and themes. The study team judged that thematic saturation occurred two-thirds into recruitment. Recruitment continued beyond this in accordance with our goal of achieving heterogeneity through purposive sampling.^22^ A summary of our findings was shared with participants for their feedback.^23^ Our reporting conforms to the Consolidated Criteria for Reporting Qualitative Studies (COREQ) reporting guideline.

## Results

We interviewed 52 participants. The median participant HbA1C at the time of interview was 7.4%. The demographic and clinical information of our participants (*n* = 52) are available in Table 1. The themes and sub-themes are summarised in Table 2.

**Table 1.** Demographic and clinical information of participants (*n* = 52)

**Median age, years (IQR)**	58 (12.3)
**Sex,** ***n*** **(%)**	
Male	30 (57.7)
Female	22 (42.3)
**Ethnic group,** ***n*** **(%)**	
Chinese	24 (46.2)
Indian	14 (26.9)
Malay	8 (15.4)
Others	6 (11.5)
**Educational level,** ***n*** **(%)**	
No formal education	1 (1.9)
Primary	3 (5.8)
Secondary or Institute of Technical Education	20 (38.5)
GCE 'A' level or International Baccalaureate or polytechnic diploma	16 (30.8)
University and above	12 (23.1)
**Marital status,** ***n*** **(%)**	
Single	3 (5.8)
Married	41 (78.9)
Divorced	3 (5.8)
Widowed	5 (9.6)
**Housing type,** ***n*** **(%)**	
HDB 1–2 room flat	1 (1.9)
HDB 3-room flat	3 (5.8)
HDB 4-room flat	13 (25.0)
HDB 5-room or executive flat	34 (65.4)
Condominium or landed property	1 (1.9)
**Polyclinic, *n* (%)**	
Pioneer	36 (69.2)
Jurong	16 (30.8)
**Number of CSP conversations**	
1	26 (50.0)
2	22 (42.3)
3	4 (7.7)
**Median years living with diabetes mellitus (IQR)**	9 (7)
**Median Hba1c (IQR)** ***most recent reading before interview***	7.4 (1.6)
< 7% (*n*, %)	19 (36.5)
≥7% (*n*, %)	33 (63.5)
**Diabetes management,** ***n*** **(%)**	
Insulin-requiring	10 (19.2)
Non-insulin-requiring	42 (80.8)
**Comorbidities, *n* (%)**	
None	1 (1.9)
Hypertension	3 (5.8)
Hyperlipidaemia	12 (23.1)
Hypertension and hyperlipidaemia	36 (69.2)

CSP = care and support planning. HDB = Housing & Development Board; refers to Singapore's public housing authority and a statutory board under the Ministry of National Development. IQR = interquartile range.

**Table 2.** Themes and sub-themes

Theme	Sub-themes
The importance of the care-planning letter (CPL)	A reflective promptPreparation for the personalised care and support planning (CSP) conversationThe challenge of language proficiency
The role of the programme coordinator	Ensured smooth running of programmeProgramme coordinator as advocateComing alongside with empathy
The value of the CSP conversation	Healthcare provider as listenerTime for relational communicationPractitioners and patients as partners
Agency in self-management	Care ownership: from provider to patientCare goals: from abstract to concreteAgency takes time

All quotes are verbatim and include Singaporean English (Singlish) expressions, which may appear unusual to readers accustomed to British English.^24^ The expression '*lah*' punctuates Singlish speech to indicate emphasis. Some edits in the quotes indicated by parentheses [...] have been added to increase clarity for the reader. We have included additional quotes to support the themes and sub-themes in the Supplementary Table S2.

The main themes and sub-themes demonstrate how people living with diabetes experience the different elements within PACE-D, ranging from the experience of preparing for the consultation with the CPL to developing agency and creating goals for themselves.

### The importance of the care-planning letter

The CPL in the form of a handy booklet that summarised the latest investigation results as coloured charts were mailed to PACE-D patients 2 weeks before their CSP appointment.

#### A reflective prompt

Instead of *'just a printout' (P26*), which was how patients described investigation results printed within typical consultations, words such as *'eye-opener'* and *'visual impact' (P11*) were used to describe the CPL. For some, the CPL prompted reflection on *'… what is it that can be improved, what is it that could have gone wrong' (P49),* and sometimes nudged patients beyond mere contemplation onto the brink of action,^25^ *' ... it already is … a thought-provoking instrument … for someone to think and be serious about wanting to get in shape' (P26*).

#### Preparation for the personalised CSP conversation

The CPL prepared patients who engaged with it for more effective CSP conversations.The results were a *'talking point'* (P26) that spurred important discussion topics without need for *'beating* [around] *the bush'* (P31). The CPL meant that the participants could consider their answers:

'*… when I* [am] *with the doctor* [and hear] *some of the questions that they asked, I think I am quite ready with the answer because I thought through it.'* (P49)

With the CPL on hand, patients could enter more deeply into the CSP conversation and therefore more deeply into their own self-care:

*'Once upon a time … only the doctor* [was] *looking at the screen* [with the results] *… But since you have* [the CPL] *in your hand and you receive it beforehand … you are ready, prepared to ask him some hard question*[s]*.'* (P31)

Being CPL-equipped shifted the provider–patient dynamic in favour of the patient; they now had a greater voice in the CSP process.

#### The challenge of language proficiency

In linguistically diverse Singapore, the CPLs were made available in English, Malay, and Mandarin Chinese. Nevertheless, language proficiency often challenged patient engagement with the CPL, and thereby with CSP itself. Given both spoken and written language proficiency may be related to culture and education levels, we provide the participants’ cultural group and highest level of education achieved with the quotes in this section:

*'I asked myself, "How do I go about filling this* [CPL]*?" I did not have the confidence … I don’t know how to.*' (P30, Chinese female, secondary school)

Patients who lacked confidence with the written language relied on family members, and often on the programme coordinator to read and to make entries in the CPL on their behalf:

*'… old people face a lot of problems* [laughs]*. They have a hard time to express, like me too.'* (P25, Malay female, polytechnic diploma)

Patients reported that their confidence increased with each CSP cycle:

*'… the first time when I get the form you sent me … I still don’t really understand. The second time, I understand better. The third time also understand a bit more.'* (P41, Chinese female, primary school)

### The role of the programme coordinator

The roles of the programme coordinator beyond administrative matters surfaced as an important theme in our study.

#### Ensured smooth running of programme

The programme coordinator’s main role was to ensure that PACE-D operations would occur smoothly despite the busyness of each day at the polyclinic. This included scheduling appointments, preparing CPLs to be mailed off, linking patients with social care activities, as directed by the CSP practitioner, and re-printing the CPL when needed:

*'I told* [the programme coordinator] *I didn’t bring* [the CPL]*. I forgot so she … print out one more time.'* (P02)

#### Programme coordinator as advocate

Beyond administrative duties and merely taking *'a quick glance'* (P24) at the results booklet, the programme coordinator was noted for encouraging patients to actively engage with the CPL. This patient describes being assisted by the programme coordinator before the CSP conversation:

*'*[The programme coordinator] *go through the* [CPL] *with me and tell me about my results. I … tell her that I want to exercise more, but I don’t know how to do it indoors to make myself burn out the calories … she just writes* [this concern] *down for me* [on the CPL booklet, so that] *later* [I] *can ask the doctor lor.*' (P36)

The programme coordinator’s role was described in terms of a supporter and advocate:

*'*[The programme coordinator] *… actually gave me … a kick in the ass, to let me think* [about] *why it* [the results] *became like this or how you should do to improve, and how to improve it* [so that I] *can check with the doctor* [later]*.'* (P33)

As a result, some patients were better prepared for the personalised CSP conversations that they *'got a lot* [more out of my conversation] *with the doctor'* because *'normally I won’t talk about anything'* (P47).

#### Coming alongside with empathy

Beyond coordination and facilitation of CSP engagement, programme coordinators provided a safe, empathetic space where authentic concerns were aired:

*'So, … you know just open up to* [the programme coordinator] *as well. … I just throw it out actually like I, I don't feel like taking medication* [small laugh] *… I think she can empathise … it’s a totally different perspective from the doctors … the doctor he’s health-trained, professionally trained, so he can look at the impact that your decision how it will affect you. …* [pause]*, the PACE-D staff is more a like er supplementary role to help the doctor in helping the patient improve on the health through the build-up of community, social* [pause] *activities … togetherness.'* (P10)

This made a difference in how patients engaged in the programme. Having *'someone there to care for my health and … taking the effort …'* encouraged patients to *'put in effort in the whole programme'* (P49).

### The value of the personalised care and support planning conversation

The personalised CSP conversation was designed to be a meeting of experts: the CPL-equipped and prepared patient, and the trained CSP practitioner.

#### Healthcare provider as listener

Having been listened to in the CSP conversation was appreciated by participants. They observed that unlike 'usual' consultations, CSP practitioners who could be a family physician, nurse care manager, or pharmacist, patiently listened to their illness narrative during CSP:

*'*[The CSP practitioner] *has a lot of patience. He will explain things to you. Some doctors … just write, write, write, write, write okay, done, then did not say much. But this particular doctor did share with me a lot in different areas lah.'* (P30)

This patient appreciated how this CSP practitioner took time to listen:

*'… the doctor was very good la, very good ah, he wanted to listen, he wanted to listen.'* (P25)

This yielded dividends in terms of greater patient engagement:

*'I will be more open up to ask, more open up to find out how I can control better.'* (P24)

#### Time for relational communication

Consultation time set aside for the CSP allowed more constructive conversations to occur:

*'… if the time is too short, we will sometimes forget what to ask.'* (P41)

The consultation ceased to feel rushed:

*'*[The CSP] *was more thorough than my normal you know, hello–goodbye … now I’m a familiar face to them … the doctor was not in a rush to see me.'* (P47)

Even when pressed for time, practitioners managed somehow to not rush the patient: *'At least he did not give me the sense that he’s rushing*', this made the consultation feel *'a little more personal'* and *'if I have a question, he has time to answer*' (P49).

#### Practitioners and patients as partners

With more time for listening, most patients found the personalised CSP conversation to be less *'instructional'* and more *'participatory' (P22*). As described:

*'… there’s dialogue and OK because we discussed, he nudged me*, [at first] *I resist a little bit but* [later I] *gradually opened up … it was a friendly thing … he was offering suggestions, but leaving a lot of suggestions to me.'* (P26)

The patient–provider power dynamic was also observed to be more balanced:

*'… the consultation* [has] *now really change*[d]*, where it’s a 2-way traffic …*' (P21)

This affected the patients' perceptions of the CSP practitioner, with some describing the practitioner '… *like a friend' (P16*). Others used the words *'personal'* and *'partners'* who jointly worked towards patient goals:

*'… the whole consultation was more … professional but it’s personal … The other one* ['usual' consultation] *is very professional … very data based. This one* [personalised CSP] *is very professional but* [also] *personal …me and the doctors are partners in trying to reduce or take care of my health.'* (P49)

### Agency in self-management

Personalised CSP recognised that for patients to live well with their condition, they had to develop greater agency in diabetes self-management.

#### Care ownership: from provider to patient

Participants reported working harder to identify personalised health goals compared with usual consultations:

*'Actually, it came more from me, but the doctor did advise … I did set quite a realistic figure* [numerical target], *so she* [was] *actually just try*[-ing] *to give me some ideas rather than giving me or setting the target for me.*' (P33)

Health and sometimes aspirational life goals set by patients rather than by providers resulted in ownership of health outcomes:

*'But now is totally different … I saw my result and if I really want to achieve* [this target]*, I must follow what I promise … I’m the one who set this … I’m the one* [laugh] *not, no, no longer the doctor.'* (P21)

#### Care goals: from abstract to concrete

Staking a personal claim in outcomes resulted in goals that were more realistic, concrete, and achievable: *'… because the goals are set by ourselves … you got to be realistic ah …' (P29*).

Participants shared how initial goals written into CPL often became more concrete through the CSP conversations:

*'I … hopefully will bring down the weight … we were looking at it and then* [the CSP practitioner said] *how much of it are you going to reduce? I say I put down there already …. 60 plus, So … 69 or sixty, so,* [the CSP practitioner] *wants me to put a range. Ya, I put it down, so that is the goal that I’m going lah, 65–69* [kg] *around there.'* (P16)

Writing the personalised goal was an act of ownership and agency that both excited and challenged participants:

*'… when you put something in black and white, you really want to make it happen right? It’s not something that you want to be flaky … So,* [the doctor] *say can* [you do what you have written] *or not? I say I think can ah so he says OK change* [laugh] *… there’s some form of challenge.'* (P26)

#### Agency takes time

The shift in disposition of patient from receiver of advice to being an explorer of creative solutions took time and some effort:

*'*[When asked to] *share your own ideas … hmm I usually don’t because I do not have ideas of my own, so the answer is I got a lesser chance* [of generating ideas]*. I will have to wait for the doctor to give me the information*.' (P44)

This patient shared how new plans occurred to them the weekend after the CSP conversation:

*'Umm, I, after a couple of days after the consultation, over the weekend, I suddenly said like “hey you know I got to plan something else” it started me thinking that I got to do something …'* (P44)

With increased rapport with their providers, patients could imagine how they could better generate ideas and plans for themselves:

*'I* [don’t] *know the doctor yet. If I know him or her better maybe I will* [suggest ideas]*, maybe the bond will be there but at this moment I don’t think I will.*' (P37)

So, while challenges remained, some patients could envision themselves increasing in agency and capacity for self-care as the provider–patient bond strengthened over time.

## Discussion

### Summary

In this study, participants unanimously responded positively to personalised CSP. Taken together, the main themes and sub-themes identified have demonstrated how people living with diabetes experience the different elements within PACE-D, ranging from the experience of preparing for the consultation with the CPL to creating goals for themselves. Although language proficiency affected engagement, for many the CPL prompted sometimes uncomfortable reflection on missed outcome targets. Others found in the programme coordinator an encouraging ally who provided empathy and advocacy in diabetes care beyond the call of duty. Patients found and appreciated that the CSP practitioners were notable for their unrushed attention, and for treating patients as partners in care. The combination of factors encouraged patients to engage more actively in the CSP conversation, to find their voices for what personally mattered most in living well with diabetes, and over time to increase in agency in diabetes self-management.

### Strengths and limitations

The qualitative interview methodology allowed us to have an in-depth understanding of the patient perspective of PACE-D (Figure 1). We interviewed 52 participants, which is a substantial number for research of this nature. As part of our purposive sampling strategy, we managed to achieve representation of the main ethnic groups (Chinese, Malay, Indian) in Singapore. Most of our participants had relatively good diabetes control (median HbA1C: 7.4%) and a higher rate of secondary and post-secondary education (92.4%), compared with the national rate (74.5%).^26^ In addition, data quality might have been affected because we needed to conduct the interviews using a mix of modalities (in-person, phone, live-streamed) amid pandemic safety measures, and one of the two interviewers had prior interactions with participants.

### Comparison with existing literature

Similar to the experience of patients in the UK,^5,9^ patients in our study felt that personalised CSP strengthened their sense of agency and allowed them to have a stake in determining their own goals. This sense of agency, however, sometimes took time to develop, and often depended on the language proficiency of patients.^27–29^ Patients appreciated that the CPL prepared them for CSP conversation^9,30^ and that the non-administrative empathetic and motivational role of the programme coordinator cohered with the literature on the positive contribution of health coaches as facilitators and advocates for people living with diabetes in primary care settings.^31^ Participants also noted that personalised CSP conversations contrasted with usual consultations in that time was intentionally set aside, providers listened better, and these fostered better patient–provider relationships. These results align with research that identified the importance of 'listening and learning' during consultations to build a 'caring relationship' over time.^32^ Two recent reviews have also highlighted how having a good patient–provider relationship improved self-management and health outcomes.^33,34^

### Implications for research and practice

Our study has pointed to the need for future research in several key areas.

First, research is needed on who benefits most from personalised CSP. We observed that patients with stronger language proficiency were generally more engaged with the CSP process. Our study participants (median HBA1c: 7.4%) generally had well controlled diabetes reflective of the Singapore polyclinic population (80% have HBA1c <8%; internal data).

As an intervention that requires careful resource planning, it matters that we ask which subgroups of people living with diabetes would benefit most from the personalised CSP process. We did not purposively sample patients based on diabetes control (for example, HBA1c level) in this study. Subsequent interventions stratifying individuals with different levels of diabetes control on their experience of personalised CSP will be instructive. In addition, providing for individuals with weaker language proficiency and possibly weaker health literacy could guide future resource allocation in the time- and staffing-constrained polyclinic context.

Second, we need to explore how we amplify agency in self-management. While trained practitioners had the explicit role as partners in the CSP conversation, the research team observed how the programme coordinator’s role mattered. In addition to administrative issues, they provided resources, nudged agency, and offered a listening ear to people living with diabetes. We are intrigued by how a dedicated and motivated *albeit* non-medically trained team member (for example, health peers, health concierges) could open up pathways in the optimal care of people living with diabetes and other LTCs.

Third, we need to research how time may be best used. Time is a scarce resource in health care. For personalised CSP, it helps to think about time 'during' and 'in between' the CSP conversation.

When thinking about time 'during' the CSP conversation, time is needed for the CSP process. In contrast to rushed 'usual consultations', participants cited how they were thankful for the time set aside for constructive discussions. They felt listened to, found a voice for their doubts and concerns, and partnered with providers in the care of their LTC.

Time is needed to develop agency. Our data has shown that repeated annual CSP conversations facilitate self-management. Insights gained during the consultation helped patients transition from mere contemplation to decisive action.

When thinking about using time 'in between' the CSP conversation, while preparation time (Figure 1) is set aside in the design of CSP process, we found receiving the CPL can be a trigger for patients. Only a small minority of patients give the letter a brief glance. Most patients derive substantial insights from it. A significant number harness the CPL to better understand the nuances of their health. Others proactively contemplate its contents, even discussing their reflections with the programme coordinator before CSP conversations. Whether immediate or deferred, the letter has a marked influence on most patients' approach to their health journey.

Research is also needed on going beyond diabetes and beyond polyclinics. Our study focused on the care for people living with diabetes, an archetypal LTC. Many of the factors required for self-management would be echoed in the management of other LTCs, for example, hypertension. Researchers see a role for CSP for the care of LTCs beyond diabetes.

In addition, personalised CSP has the potential to be extended from the public polyclinic setting to the private general practice or family practice setting, which provides an estimated 80% of primary care in Singapore.^35^

Lastly, research must consider non-English speakers. With just three participants who were interviewed in non-English languages in this study, we are unable to make conclusions about how non-English speakers experience personalised CSP. There may be value in specifically sampling Asian language speakers for their experience of CSP. The potential of personalised CSP to strengthen self-management may be an important strategy to contain rising numbers of patients with diabetes and other LTCs in Singapore, and other countries in the region.

## Data

The dataset relied on in this article is available from the corresponding author on reasonable request.
